# Comparison of Diffusion Tensor Imaging Metrics in Normal-Appearing White Matter to Cerebrovascular Lesions and Correlation with Cerebrovascular Disease Risk Factors and Severity

**DOI:** 10.1155/2022/5860364

**Published:** 2022-10-21

**Authors:** Seyyed M. H. Haddad, Christopher J. M. Scott, Miracle Ozzoude, Courtney Berezuk, Melissa Holmes, Sabrina Adamo, Joel Ramirez, Stephen R. Arnott, Nuwan D. Nanayakkara, Malcolm Binns, Derek Beaton, Wendy Lou, Kelly Sunderland, Sujeevini Sujanthan, Jane Lawrence, Donna Kwan, Brian Tan, Leanne Casaubon, Jennifer Mandzia, Demetrios Sahlas, Gustavo Saposnik, Ayman Hassan, Brian Levine, Paula McLaughlin, J. B. Orange, Angela Roberts, Angela Troyer, Sandra E. Black, Dar Dowlatshahi, Stephen C. Strother, Richard H. Swartz, Sean Symons, Manuel Montero-Odasso, ONDRI Investigators, Robert Bartha

**Affiliations:** ^1^Centre for Functional and Metabolic Mapping, Robarts Research Institute, University of Western Ontario, London, Canada; ^2^L.C. Campbell Cognitive Neurology Research Unit, Hurvitz Brain Sciences Research Program, Sunnybrook Research Institute, Toronto, Ontario, Canada; ^3^Department of Medicine (Neurology), Sunnybrook Health Sciences Centre and University of Toronto, Toronto, Canada; ^4^Clinical Psychology, University of Toronto, Toronto, Canada; ^5^Clinical Neurosciences, University of Toronto, Toronto, Canada; ^6^Rotman Research Institute, Baycrest Health Sciences, Toronto, Canada; ^7^Dalla Lana School of Public Health, University of Toronto, Toronto, Canada; ^8^Thunder Bay Regional Health Research Institute, Thunder Bay, Canada; ^9^Queen's University, Kingston, Canada; ^10^Department of Medicine, University of Toronto, Toronto, Canada; ^11^Department of Medicine, Division of Neurology, University of Western Ontario, London, Canada; ^12^Department of Medicine, Faculty of Health Sciences, McMaster University, Hamilton, Canada; ^13^Li Ka Shing Knowledge Institute, Toronto, Canada; ^14^Thunder Bay Regional Research Institute, Thunder Bay, Canada; ^15^School of Communication Sciences and Disorders, Western University, London, Canada; ^16^Roxelyn and Richard Pepper Department of Communication Sciences and Disorder, Northwestern University, Evanston, USA; ^17^Sunnybrook Health Sciences Centre, University of Toronto, Stroke Research Program, Toronto, Canada; ^18^Ottawa Hospital Research Institute, Ottawa, Canada; ^19^Department of Medical Biophysics, University of Toronto, Toronto, Canada; ^20^Department of Medical Imaging, Sunnybrook Health Sciences Centre, Toronto, Canada; ^21^Department of Medicine, Division of Geriatric Medicine, Parkwood Hospital, St. Joseph's Health Care London, London, Canada; ^22^Ontario Neurodegenerative Disease Initiative, Ontario Brain Institute, Toronto, Canada; ^23^Department of Medical Biophysics, University of Western Ontario, London, Canada

## Abstract

Alterations in tissue microstructure in normal-appearing white matter (NAWM), specifically measured by diffusion tensor imaging (DTI) fractional anisotropy (FA), have been associated with cognitive outcomes following stroke. The purpose of this study was to comprehensively compare conventional DTI measures of tissue microstructure in NAWM to diverse vascular brain lesions in people with cerebrovascular disease (CVD) and to examine associations between FA in NAWM and cerebrovascular risk factors. DTI metrics including fractional anisotropy (FA), mean diffusivity (MD), axial diffusivity (AD), and radial diffusivity (RD) were measured in cerebral tissues and cerebrovascular anomalies from 152 people with CVD participating in the Ontario Neurodegenerative Disease Research Initiative (ONDRI). Ten cerebral tissue types were segmented including NAWM, and vascular lesions including stroke, periventricular and deep white matter hyperintensities, periventricular and deep lacunar infarcts, and perivascular spaces (PVS) using T_1_-weighted, proton density-weighted, T_2_-weighted, and fluid attenuated inversion recovery MRI scans. Mean DTI metrics were measured in each tissue region using a previously developed DTI processing pipeline and compared between tissues using multivariate analysis of covariance. Associations between FA in NAWM and several CVD risk factors were also examined. DTI metrics in vascular lesions differed significantly from healthy tissue. Specifically, all tissue types had significantly different MD values, while FA was also found to be different in most tissue types. FA in NAWM was inversely related to hypertension and modified Rankin scale (mRS). This study demonstrated the differences between conventional DTI metrics, FA, MD, AD, and RD, in cerebral vascular lesions and healthy tissue types. Therefore, incorporating DTI to characterize the integrity of the tissue microstructure could help to define the extent and severity of various brain vascular anomalies. The association between FA within NAWM and clinical evaluation of hypertension and disability provides further evidence that white matter microstructural integrity is impacted by cerebrovascular function.

## 1. Introduction

Cerebrovascular disease (CVD) contributes to several neurological disorders including vascular cognitive impairment no dementia (VCIND), vascular dementia (VaD), and mixed vascular/neurodegenerative dementia. Vascular cognitive impairment (VCI) remains the second leading cause of dementia after Alzheimer's disease (AD) accounting for 20% of dementia cases [1, 2]. Moreover ~30% of stroke patients also develop dementia [3] which may be classified as VCI. In all cases, early diagnosis requiring the identification of CVD and precise prediction of disease progression remain among the most important clinical needs for appropriate management [1, 4]. Identification of diverse vascular lesions by magnetic resonance imaging currently requires assessment of numerous imaging contrasts. Improved detection of vascular lesions and tissue microstructural changes in normal-appearing tissue would enhance the early identification of VCI.

VCI pathogenesis is associated with cerebrovascular anomalies in both white matter (WM) and grey matter (GM) including stroke and ischemic lesions, lacunar infarcts, and microhemorrhages [4]. Clinical assessments of these lesions are typically performed by visualization using noninvasive multimodal neuroimaging techniques such as magnetic resonance imaging (MRI) [5, 6]. By creating a wide range of contrasts, MRI has the capacity to identify diverse ischemic tissue anomalies. For instance T_1_- and T_2_-weighted images can typically detect large hemorrhages as well as large chronic stroke lesions [7], and T_2_^∗^-weighted images can identify microhemorrhages [7]. Fluid-attenuated inversion recovery (FLAIR) can differentiate complete and incomplete infarcts as hypointense and hyperintense regions, respectively [8]. Furthermore, WM pathological alterations such as WM hyperintensities (WMHs) and leukoaraiosis can be identified in diffusion-weighted images (DWIs) and FLAIR [4, 8]. However, despite the use of these advanced methods to detect vascular disease, both sensitive and specific neuroimaging biomarkers of VCI are still lacking [5, 6].

Diffusion tensor imaging (DTI) measures of fractional anisotropy (FA) and mean diffusivity (MD) provide a high level of sensitivity to tissue microstructure in comparison with other MRI techniques. Interestingly, FA in normal-appearing WM (NAWM) has been shown to be sensitive to microstructural abnormalities in several conditions [9–11] including vascular disease and stroke, in people with cognitive impairment [12–17], and can vary with location in the brain [18]. Variations in DTI measures, particularly FA [19–22]) in NAWM, may be valuable prognostic indicators of cognitive decline as these metrics correlate more with cognition than other MRI measures [23, 24]. DTI measures are also sensitive to tissue damage following acute cerebral ischemic injuries, which can help with understanding cognitive changes following acute stroke [25], subcortical ischemic vascular disease [26], and cerebral ischemia following subarachnoid hemorrhage (SAH) [27].

Although conventional DTI metrics (FA, MD, axial diffusivity (AD), and radial diffusivity (RD)) are sensitive to many vascular pathologies, these measures may not be ideal indicators of tissue microstructure in brain regions where the single compartment diffusion tensor model does not adequately represent tissue microcompartments and their arrangements [28–30]. Considering this limitation and the substantial variability in the location and size of cerebrovascular anomalies, the purpose of this study was to determine whether conventional DTI metrics, which are commonly incorporated into routine clinical evaluations, differed in diverse chronic ischemic anomalies, vascular lesions, and major cerebral tissue types including NAWM, normal-appearing GM (NAGM), and cerebrospinal fluid (CSF). Ischemic lesions included chronic stroke lesions, periventricular WMHs (pWMHs), deep WMHs (dWMHs), and perivascular spaces (PVSs), as well as periventricular lacuna (pLACN), and deep lacuna (dLACN). Brain tissue characterization that incorporates DTI metrics could improve the sensitivity and specificity of lesion detection. The secondary objective of this study was to determine whether FA as a measure of microstructural integrity in NAWM correlated with the concentration of major blood analytes associated with CVD, blood pressure, and the presence of CVD risk factors. We hypothesized that FA in NAWM, which represents disruptions in underlying tissue microstructure, would be associated with CVD risk factors.

## 2. Materials and Methods

### 2.1. Participants

One hundred and fifty-two participants from the Ontario Neurodegenerative Disease Research Initiative (ONDRI) with CVD and complete imaging datasets were included. ONDRI is a provincial collaborative longitudinal research program in Ontario to study diverse neurodegenerative diseases such as Alzheimer's, Parkinson's, and CVD [31–33]. The study was approved by the Ethics Review Boards at all participating institutions [32]. Specific inclusion criteria for the ONDRI study have been previously described [32]. Inclusion in the CVD cohort included age between 55 and 85 years, an ischemic stroke event documented by MRI or CT, ≥3 months since stroke, modified Rankin scale (mRS) 0-3 (except one participant who had an mRS of 4), and a history of baseline dementia with prestroke modified Rankin score ≤ 2. Presence of previous silent strokes was allowed [32]. Exclusion criteria included no vascular cause of symptoms, large cortical strokes > 1/3 middle cerebral artery territory, severe cognitive impairment, aphasia, inability to write, and/or severe functional disability limiting ability to perform assessments.

Five major CVD risk factors were documented for the study participants: (1) diabetes, (2) hypertension (HTN), (3) high cholesterol or hypercholesterolemia (HCL), (4) coronary artery disease (CAD), and (5) other cardiovascular conditions (such as atrial fibrillation, stenting, coronary artery bypass grafting, abnormal heart rhythms, cardiomyopathies, congenital heart disease, and heart valve disease). These clinical conditions were identified through medical histories and conditions at screening. The global cognition was also assessed in the studied subjects by the Montreal Cognitive Assessment (MoCA) (ranging from 0 to 30) (Table 1). The participants were also evaluated using the NIH stroke scale (NIHSS) [34].

### 2.2. Magnetic Resonance Imaging Data Acquisition

The acquisition of neuroimaging data for ONDRI participants has been previously described and was consistent with the Canadian Dementia Imaging Protocol (CDIP) [32]. Briefly, T_1_-, T_2_-, and T_2_^∗^-weighted, FLAIR, resting state functional MRI (fMRI), and diffusion MRI (dMRI) images were available and incorporated from 10 different 3.0 T MRI scanners across Ontario. The DTI processing pipelines utilized T_1_-weighted (voxel dimensions of 1.0 × 1.0 × 1.0 mm^3^), T_2_-weighted images (0.9375 × 0.9375 × 3.0 mm^3^), and DTI data. All the ONDRI DTI data were acquired with 30 different gradient directions (with *b* = 1000 s/mm and flip angle 90°) plus at least one *b*_0_ volume with voxel dimensions of 2.0 × 2.0 × 2.0 mm^3^.

### 2.3. Segmentation of Cerebral Tissues and Vascular Lesions

The procedures used to segment regions of interest have been detailed previously (Semiautomatic Brain Region Extraction (SABRE) [35], Lesion Explorer [36, 37], and Fuzzy Lesion EXtractor (FLEX) [38]), including a scan-rescan reliability analysis [39]. Briefly, interleaved proton density (PD) and T_2_-weighted images and FLAIR images were coregistered to the T_1_-weighted image, and a PD-T_2_-based mask was automatically generated and manually edited. Using this mask, the T_1_-weighted image was segmented using a multifeature histogram method [40] to generate GM, WM, and CSF regions, and ventricular CSF (vCSF) was manually relabeled. WMHs and lacunae were also automatically identified using FLEX (FLAIR-based) and Lesion Explorer [36, 37]. Each lesion type was further subdivided into a region-based class (periventricular or deep) by an automated algorithm [36–38]. Lesion Explorer was also used to capture enlarged PVS [37, 41] and cortical strokes were manually traced. A combination of these tissue segmentation methods produced the following ten different tissue classes: NAWM, NAGM, sulcal CSF (sCSF), vCSF, pWMH, dWMH, pLACNs, dLACNs, enlarged PVS, and strokes.

In this study, the variation of DTI metrics in these 10 different tissue/lesion classes was examined and compared. Lesion volumes < 24 mm^3^ (corresponding to at least three DTI voxels) were not considered to reduce partial volume errors. The careful segmentation of these normal and pathological tissues provided a unique opportunity to characterize the associated water diffusion metrics obtained by DTI.

### 2.4. Automated DTI Processing Pipelines

DTI data were analyzed using an automated pipeline previously described and validated [42, 43] consistent with the well-known Enhanced Neuroimaging Genetics through Meta-Analysis (ENIGMA) DTI framework [44–46]. In this pipeline, two major quality control (QC) procedures were added to the general ENIGMA DTI recommended framework to increase the reliability and precision of DTI measurements. The first QC procedure is based on the “DTIPrep” protocol [47, 48]. The second QC procedure is based on Robust Estimation of Tensors by Outlier Rejection (RESTORE) algorithm [49]. The pipeline incorporated artifact and noise removal, registration to the corresponding T_1_-weighted structural image, and tensor fitting to produce maps of FA, MD, AD, and RD in each subject.

### 2.5. ROI Statistical Analysis

The DTI scalar metric maps for FA, MD, AD, and RD, along with the cerebral tissue/lesion masks, were used to calculate the mean of the DTI metrics in the 10 cerebral tissue types and lesions previously described. The mean values of the DTI metrics were compared between brain lesions and healthy tissues to determine whether DTI metrics could be used for lesion classification and to develop complex DTI-based biomarkers for diagnostic and therapeutic purposes despite the substantial variability in the location and size of the various lesions identified in the brain.

### 2.6. Statistical Analysis

#### 2.6.1. Tissue-Based DTI Metric Variations

The mean and coefficient of variation (CV) were measured for the four major DTI metrics (FA, MD, AD, and RD) in each of the 10 different cerebral tissue types/lesions to compare DTI metric variations between these tissue types/lesions. All the region of interest (ROI) statistical analyses described below were implemented in MATLAB (R2019b, Natick, Massachusetts: The MathWorks Inc.) and IBM SPSS (Version 26.0, Armonk, NY: IBM Corp).

#### 2.6.2. Tissue/Lesion Classification Based on DTI Metrics

**Figure 1:**
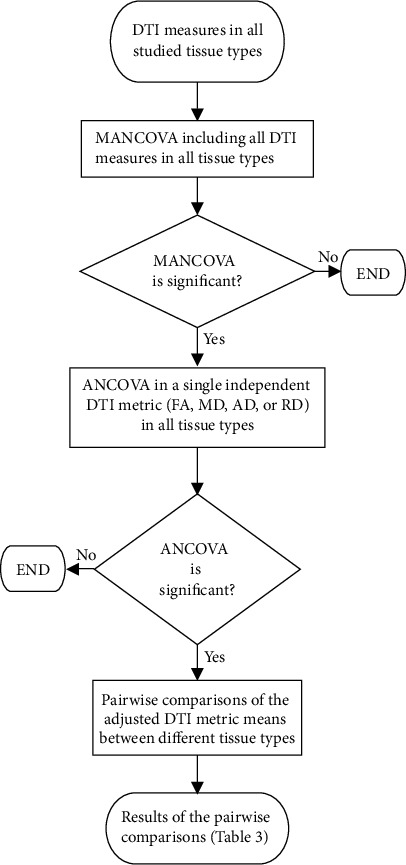
Flow chart of the statistical analyses conducted to identify DTI metric differences between tissue types.

A flowchart of the statistical tests conducted is provided in Figure 1. To identify differences in DTI metrics (FA, MD, RD, and AD) between cerebral tissues/lesions, a MANCOVA was performed including all tissue types and DTI metrics as dependent variables while adjusting for age, sex, and education level (Figure 1) [50]. When significant, MANCOVA was followed by ANCOVAs for each DTI metric to identify which DTI metrics showed differences between tissue types (Figure 1) [51]. The ANCOVA tests, similar to the MANCOVA, also were adjusted for age, sex, and education level. If significant, the estimated marginal means were utilized to perform pairwise comparisons of the adjusted DTI metric means between different tissue types. These comparisons were limited to FA and MD because (1) these are the most common DTI parameters used for tissue microstructure evaluation and (2) while AD and RD provide valuable information about WM tissue microstructure in specific brain conditions and WM anomalies, in the studied CVD population, they were highly correlated with MD, which was tested using linear regressions adjusted for age, sex, and education level. Hence, comparisons of FA and MD were considered to be sufficient. It should be noted that MANOVA and follow-up ANOVA tests were also performed without adjustment for age, sex, and education level to determine whether removing these covariates altered the results of the statistical tests. In all tests, *p* values < 0.05 were considered significant.

#### 2.6.3. Correlation between FA Measured in NAWM and Blood Analytes and Blood Pressure

FA is the DTI metric most sensitive to pathology-related microstructural alterations within NAWM [19–22]; hence, FA in NAWM was examined for associations with levels of five blood analytes (glucose, triglyceride, cholesterol, high-density lipoprotein (HDL) cholesterol, and low-density lipoprotein (LDL) cholesterol), systolic BP, and diastolic BP, using separate linear regressions adjusted for age, sex, education level, body mass index (BMI), ethnicity, smoking history, and alcohol consumption [52–55]. Previous studies have adjusted results with respect to WMH volumes and total intracranial volumes when examining DTI metrics in NAWM [55–58]. Therefore, we repeated the regression analyses between FA in NAWM and blood analytes/BP using separate linear regressions weighted by WMH volumes normalized to the total intracranial volumes (TIV). Using this approach, subjects with greater normalized WMH volume had a proportionally greater influence on the linear regression model [59, 60]. These weighted linear regressions were also adjusted for age, sex, education level, BMI, ethnicity, smoking history, and alcohol consumption. In our analyses, significant correlations were identified by *p* values < 0.0071 (significance level was defined as *α* = 0.05 divided by 7 tests) after the Bonferroni correction for multiple comparisons.

#### 2.6.4. FA Measured in NAWM and CVD Risk Factors

FA in NAWM was also evaluated in association with five major CVD risk factors (diabetes, HTN, HCL, CAD, and other cardiovascular conditions), using ANCOVA. For each CVD risk factor, ANCOVA was conducted to compare FA in NAWM in subjects with the specific CVD risk factor to that in subjects without the specific CVD risk factor. These comparisons were also repeated when weighting the FA measurements in NAWM by the WMH volumes normalized to the TIV to increase the influence of subjects with greater WMH volume. All ANCOVAs were adjusted for age, sex, education level, BMI, ethnicity, smoking history, and alcohol consumption. In our analyses, significant difference between the groups were identified by *p* values < 0.01 (significance level is defined as *α* = 0.05 divided by 5 tests) after the Bonferroni correction for multiple comparisons.

#### 2.6.5. Association between FA Measured in NAWM and Clinical Scales

Similarly, FA in NAWM was evaluated in association with mRS as well as NIH stroke scale (NIHSS). The mRS is a measure of the level of disability or dependence in stroke patients or people with other neurological disorders [61]. The NIHSS is an overall measure of impairment caused by stroke considering various aspects of brain function such as language, sensory, and motor performance [34]. ANCOVA was used to compare FA in NAWM in subjects as a function of mRS score (0, 1, 2, 3, and 4 where higher scores indicate more severe disability). ANCOVA was also used to compare FA in NAWM in subjects as a function of three NIHSS ranges (0 indicating no stroke symptoms, 1-4 indicating minor stroke symptoms, and 5-6 indicating moderate stroke symptoms). If the ANCOVA was significant, the estimated marginal means were utilized to perform pairwise comparisons of the adjusted mean FA in NAWM between the five groups of subjects with different mRS scores (0, 1, 2, 3, and 4) or the three groups of subjects with different NIHSS ranges. The ANCOVA was also repeated when weighting the FA in NAWM by the WMH volume normalized to the TIV. If significant, similar pairwise comparisons between the adjusted mean FA in NAWM in the five groups with different mRS scores or the three groups with different NIHSS ranges were conducted. The ANCOVA tests were adjusted for age, sex, education level, BMI, ethnicity, smoking history, and alcohol consumption. In our analyses, significant difference between the groups were identified by *p* values < 0.05.

## 3. Results

### 3.1. Participants

CVD subjects included in this study were aged 55-85 years: 31% female, with 28.3 ± 4.3 (mean ± standard deviation (SD)) (kg/m^2^) BMI, with 14.6 ± 2.9 (mean ± SD) education level, 54% with a smoking history of at least three months, 67% with alcohol consumption, and 83.6% White, while the remaining subjects were from Black (5.3%), Jewish (2.0%), Hispanic (0.6%), West Asian (1.3%), South Asian (2.0%), Chinese (3.2%), Japanese (0.6%), Filipino (0.6%), and multiple ethnicities (0.6%). Prevalence of diabetes was 22%, HTN was 74%, HCL was 79%, CAD was 17%, and other cardiovascular conditions were 35%. The concentrations of blood components such as glucose, triglyceride, cholesterol, HDL cholesterol, and LDL cholesterol along with systolic blood pressure (SBP) and diastolic blood pressure (DBP) which might impact brain WM tissue integrity [55, 62–65] are provided in Table 1. Among the 152 subjects studied, 57% had stroke lesions ranging in size from small (~50 mm^3^) to very large (~158,000 mm^3^), 76% were identified as having PVSs, 37% with dLACN, and 56% with pLACN visible in their scans (Table 2). The NIHSS was zero for 57% of the subjects (indicating no stroke symptoms), ranged from 1 to 4 for 36% of the subjects (indicating minor stroke symptoms), and ranged from 5 to 6 only for two subjects (1.3% of the subjects) (indicating moderate stroke symptoms).

### 3.2. Image Processing

**Figure 2:**
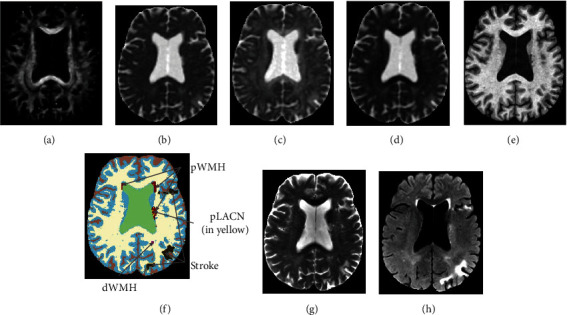
FA (a), MD (b), AD (c), and RD (d) maps produced by the DTI processing pipeline. Corresponding T_1_-weighted structural image (e), semiautomated segmentation of cerebral tissues/vascular lesions (f), T2-weighted image (g), and FLAIR image (h) are also provided. pLACN: periventricular lacuna; pWMH: periventricular white matter hyperintensities; dWMH: deep white matter hyperintensities.

Parametric maps of FA, MD, AD, and RD were successfully produced in all subjects. Typical parametric maps produced by the pipeline from one CVD subject are provided in Figures 2(a)–2(d), along with the corresponding T1-weighted image (Figure 2(e)). Several tissue types are visible in this example including stroke lesions, dWMH, pWMH, and pLACN (Figure 2(f)). Corresponding T2-weighted and FLAIR images are also provided in Figures 2(g) and 2(h), respectively. In the FA map (Figure 2(a)), all the vascular abnormalities including stroke, dWMH, pWMH, and pLACN are present as conspicuous dark regions with lower FA compared with surrounding NAWM. In the MD (Figure 2(b)), AD (Figure 2(c)), and RD (Figure 2(d)) maps, the tissue anomalies including stroke, dWMH, pWMH, and pLACN appear brighter compared with neighbouring tissue potentially indicating axonal sheath disruptions, edema, or cell death [66–73].

### 3.3. Tissue-Based DTI Metric Variations

**Figure 3:**
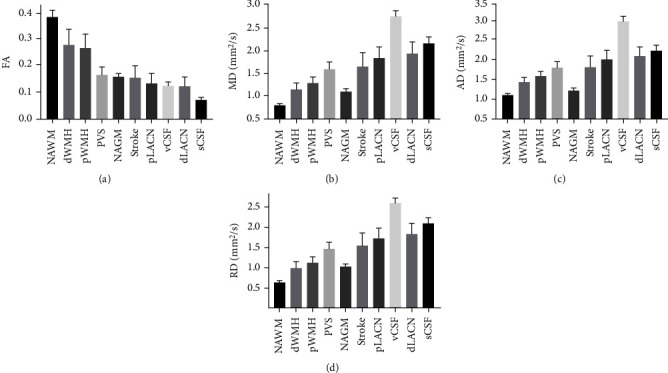
The mean FA (a), MD (b), AD (c), and RD (d) values measured by the DTI processing pipeline are provided for all segmented tissues. Error bars represent the coefficient of variation.

All studied anomalies including stroke lesions, dWMH, pWMH, dLACN, pLACN, and PVS were heterogeneously distributed among the cohort. For instance, 43% of subjects did not have a stroke lesion, while in the remaining 57% of subjects who had stroke lesions, the size of the lesions varied from very small (~50 mm^3^) to very large (~158,000 mm^3^). The locations of the stroke lesions were also variable with strokes located in different brain regions across the cohort. All other vascular lesions displayed a similar heterogeneity. The mean FA is provided in descending order for each tissue type (Figure 3(a)) along with measurement precision indicated by the error bars representing the coefficient of variation (CV). Similarly, Figures 3(b)–3(d) show the mean MDs, ADs, and RDs and corresponding CV in the different tissues. The mean DTI metric values, CV, and 95% confidence interval (CI) are also provided in Table 2.

### 3.4. Tissue/Lesion Classification Based on DTI Metrics

**Figure 4:**
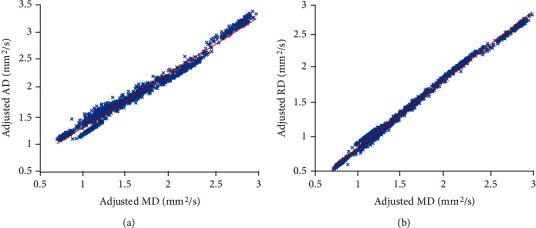
Scatter plots showing the correlation between MD and AD (a) (*p* value < 0.001, *r* = 0.990) as well as MD and RD (b) (*p* value < 0.001, *r* = 0.998).

**Figure 5:**
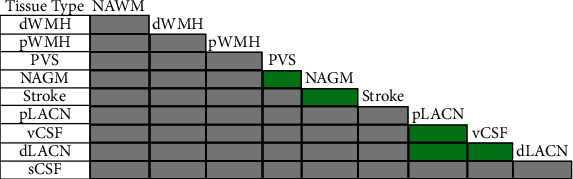
Differences in the FA and MD values measured by the DTI processing pipeline between the tissue types considered in this study. Cells shaded with gray indicate significant difference (*p* value < 0.05) between both FA and MD values in the two related tissue types. Cells shaded with green indicate significant differences (*p* value < 0.05) in MD but not FA between tissue types. All the pairwise comparisons were adjusted for age, sex, and education level.

There were significant differences between the DTI measures of the different tissues (*p* value of the MANOVA was <0.001 with 36 degrees of freedom (DF)). Follow-up ANCOVA showed that each DTI metric (FA and MD) varied significantly between tissue types (*p* value < 0.001 with 9 DF). Linear regressions adjusted by age, sex, and education level showed significant correlation between MD and AD (*p* value < 0.001, *r* = 0.990, DF = 4, Figure 4(a)) as well as MD and RD (*p* value < 0.001, *r* = 0.998, DF = 4, Figure 4(b)) with all tissue types included. Hence, AD and RD were excluded from the pairwise comparisons of the adjusted DTI metric means. These pairwise comparisons in the different tissue types also showed significant differences (*p* value < 0.05) in FA and MD values between all tissue types, with 45 paired comparisons out of a possible 45 showing significant difference in their FA and/or MD values (Figure 5). MANOVA and follow-up ANOVA tests without adjustments for age, sex, and education level also showed significant differences between the DTI metrics in the studied tissue types (*p* value < 0.001).

### 3.5. Correlation between FA Measured in NAWM and Blood Analytes and Blood Pressure

There were no significant correlations identified between FA in NAWM and blood analytes or blood pressure. Linear regression results (*p* value, standardized coefficient *β*, and DF) between FA in NAWM and blood analytes as well as blood pressure were as follows: glucose (*p* value = 0.352, *β* = −0.77, DF = 8), triglyceride (*p* value = 0.507, *β* = 0.056, DF = 8), cholesterol (*p* value = 0.668, *β* = 0.036, DF = 8), HDL cholesterol (*p* value = 0.404, *β* = −0.077, DF = 8), LDL cholesterol (*p* value = 0.524, *β* = 0.054, DF = 8), SBP (*p* value = 0.297, *β* = −0.089, DF = 8), and DBP (*p* value = 0.482, *β* = −0.059, DF = 8). After the Bonferroni correction, no significant correlations were identified. When linear regression was weighted by normalized WMH volumes, correlations (*p* value, standardized coefficient *β*, and DF) between FA in NAWM and blood analytes as well as blood pressure were as follows: glucose (*p* value = 0.765, *β* = 0.025, DF = 8), triglyceride (*p* value = 0.291, *β* = 0.088, DF = 8), cholesterol (*p* value = 0.597, *β* = 0.044, DF = 8), HDL cholesterol (*p* value = 0.594, *β* = −0.048, DF = 8), LDL cholesterol (*p* value = 0.572, *β* = 0.047, DF = 8), SBP (*p* value = 0.727, *β* = −0.028, DF = 8), and DBP (*p* value = 0.237, *β* = −0.101, DF = 8). After the Bonferroni correction, no significant correlations were identified.

### 3.6. FA Measured in NAWM and Presence of CVD Risk Factors

**Figure 6:**
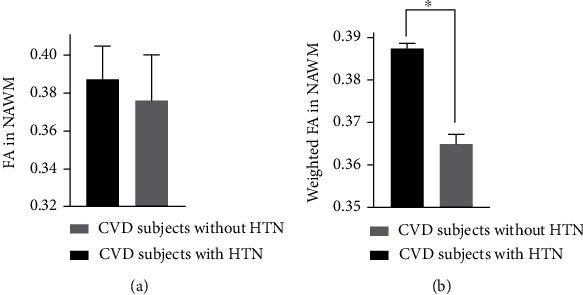
FA in NAWM in CVD subjects without and with hypertension (HTN) (a) and FA in NAWM weighted by the normalized WMH volumes in CVD subjects without and with HTN (b). The bars and error bars in panel (a) represent the mean and standard deviation of FA in NAWM. The bars and error bars in panel (b) represent the weighted mean and weighted standard deviation of FA in NAWM following statistical weighting by the normalized WMH volumes. Data with asterisk were significantly different as detected by ANCOVA test (*p* value < 0.001).

Considering FA in NAWM, there were no significant differences in people with diabetes, HTN, CAD, or other cardiovascular risk factors. However, when FA in NAWM was weighted by normalized WMH volumes, ANCOVA results (*p* value and DF) showed that FA in NAWM was lower in people with HTN (*p* value = 0.001, DF = 8) and remained significant after the Bonferroni correction. Specifically, CVD subjects with HTN had significantly lower FA in NAWM (2.9% and 5.8% when FA was weighted by normalized WMH volumes) compared to those without HTN (Figure 6).

### 3.7. Association between FA Measured in NAWM and Clinical Scales

**Figure 7:**
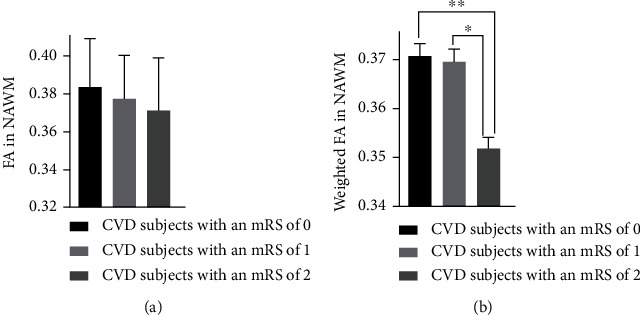
FA in NAWM in CVD subjects with various mRS scores (0, 1, and 2) (a) and FA in NAWM weighted by the normalized WMH volumes in CVD subjects with different mRS scores (0, 1, and 2) (b). The bars and error bars in panel (a) represent the mean and standard deviation of FA in NAWM. The bars and error bars in panel (b) represent the weighted mean and weighted standard deviation of FA in NAWM following statistical weighting by the normalized WMH volumes. Data with asterisk and double asterisk were significantly different as detected by pairwise comparison following the ANCOVA test with *p* value < 0.002 and *p* value < 0.001, respectively.

There were no differences in FA in NAWM between subjects with different mRS scores (*p* value = 0.193, DF = 11). However, when the FA measurements in NAWM were weighted by normalized WMH volumes, ANCOVA showed a significant group effect (*p* value = 0.006, DF = 11). Pairwise comparisons of the adjusted mean FA in NAWM between subjects with different mRS scores showed that CVD subjects with an mRS of 2 (*N* = 38) had significantly lower FA in NAWM in comparison with the CVD subjects with an mRS of both 0 (*N* = 45, *p* value = 0.001) and 1 (*N* = 64, *p* value = 0.002). The FA differences in NAWM between CVD subjects with an mRS of 0 and 2 were 3.2% and 5.1% when the FA measurements were weighted by normalized WMH volumes, while the FA differences between CVD subjects with an mRS of 1 and 2 were 1.6% and 2.4% when the FA measurements were weighted by normalized WMH volumes (Figure 7). No other significant difference was identified by pairwise comparisons. There were no differences in FA in NAWM between subjects with different NIHSS scores.

## 4. Discussion

The aim of the current study was to provide a comprehensive comparison of the variation of DTI metrics in diverse cerebral vascular lesions to NAWM. Vascular lesions included measures in dWMH, pWMH, and PVS, which have been only sparsely reported previously [74, 75], as well as dLACN and pLACN, which have not been previously examined separately. These measurements were completed using a DTI processing pipeline previously designed for the quantification of multisite DTI data [42]. The DTI processing pipeline successfully produced high-quality DTI parametric maps in 152 subjects with cerebrovascular disease participating in the ONDRI study. FA and MD were found to be significantly different in vascular lesion types and healthy tissue. The secondary objective was to examine the link between FA in NAWM and CVD risk factors. Interestingly, when the FA in NAWM was weighted by white matter hyperintensity volumes, this metric was lower in people with hypertension, a major CVD risk factor. Similarly, when the FA in NAWM was weighted by white matter hyperintensity volumes, it was also lower in people with a modified Rankin scale (mRS) score of 2 (slight disability) compared to people with an mRS score of both 0 (no symptoms) and 1 (no significant disability, despite some symptoms).

The measured values of FA, MD, RD, and AD were within the ranges of values reported in the literature [66–73] where available. For instance, the current study measured FA to be ~0.38 in NAWM (Figure 3(a) and Table 2). This result is consistent with the mean FA of 0.39 previously reported in the corpus callosum of stroke patients [68] and 0.34 previously reported in NAWM in aged brains [71]. The lowest FA values observed in the current study were in vCSF and sCSF as expected due to the isotropy of water diffusion in these regions [19, 76]. Similar FA values were observed in NAGM, PVS, and stroke lesions. Consistent with previous measurements, FA values in dWMH and pWMH were slightly lower than NAWM. The mean FA in WMH in aging brains was previously measured as 0.30 [70, 77], consistent with our results of ~0.27 in dWMH (Figure 3(a)). Another study also reported ~27% decrease in FA in WMH compared to NAWM in in situ postmortem brain specimens [78] consistent with our findings showing ~29% decrease in FA in WMHs (considering both dWMH and pWMH) compared to NAWM. Previous studies have also found low FA in the corticospinal tract caused by stroke lesions [66, 67]. In the current study, FA was measured as ~0.15 in stroke lesions (Figure 3(a)), consistent with the FA value previously reported as 0.18 [69]. Finally, the mean FA in GM in older individuals with small vessel disease was previously reported as 0.17 [79], in agreement with the current results of ~0.16 in NAGM (Figure 3(a)).

The pattern of change across tissue types observed in MD was very similar for AD and RD (Figures 3(b)–3(d)) which was confirmed by linear regression (Figure 4). The minimum values of MD and RD were observed in NAWM as expected [80, 81]. MD values of ~0.79 in NAWM and ~1.14 in dWMH (Figure 3(b)) were consistent with the MD in NAWM of 0.78 and the MD of 1.00 in WMH of people with minor stroke [71]. Mean MD in GM in older individuals with small vessel disease was also previously reported as 1.15 [79], which is slightly higher than our results of ~1.09 for MD in NAGM (Figure 3(b)). Values of AD were similar to RD and MD except that a greater difference was observed between dWMH and NAGM, where NAGM showed even lower AD. In stroke lesions, MD measured in the current study was ~1.64 (Figure 3(b)) slightly lower than the value of 1.83 previously reported [69]. Stroke lesions, pWMH, and dLACN also appeared as brighter areas (with higher diffusivity) in MD, AD, and RD maps in comparison with surrounding NAWM regions [68–70, 72]. The high diffusivity and low anisotropy in these regions are likely related to the disruption of WM tracts and microstructural barriers to water diffusion in these tissues.

While MD was significantly different in all studied tissue types, FA did not differ significantly when comparing NAGM and stroke, PVS and NAGM, dLACN and pLACN, and pLACN and vCSF. Differences were observed in MD between these tissues but not FA due to the higher CV in FA measurements (Figure 3) suggesting that FA measurements are either more susceptible to measurement error or more sensitive to biological variation. The observed variance in DTI metric measurements has many potential sources including tissue heterogeneity, tissue partial volume, and differences related to the acquisition of images on different MRI systems. The higher variability may also be due to the mathematical description of FA, making it more sensitive to the heterogeneity of tissue microstructure and pathological alterations [19].

When examining the link between FA in NAWM and cardiovascular risk factors, significantly lower FA in NAWM was observed in CVD subjects who had hypertension compared to those who did not have hypertension when the FA measurements were weighted by the normalized WMH volumes (Figure 6(b)) in the statistical analysis. Previous studies have identified associations between hypertension and neurodegeneration and dementia [62, 64], lower brain volume and higher WMH volumes [52], and WM microstructural disintegration as assessed by DTI [55, 82]. It is important to note that pixels identified as WMHs were excluded from the NAWM FA measurements. Also, the volumes of WMHs were quite heterogenous in the studied CVD cohort ranging from 74 mm^3^ to 84099 mm^3^. Therefore, the FA measurements in NAWM were statistically weighted by the WMH volume to amplify the influence of participants with a greater WMH burden on the linear regression model [55–58]. Our findings suggest that microstructural disintegration of *normal-appearing* WM is evident in people with hypertension when statistically weighting the FA measurements by the normalized WMH volume.

There was also a significant difference in the weighted mean FA in NAWM (weighted by the normalized WMH volume) when comparing between CVD subjects with various degrees of disability as evaluated by the mRS score. However, after post hoc pairwise comparisons, only subjects with an mRS of 2 (slight disability) had lower FA in NAWM than subjects with an mRS of both 0 (no symptoms) and 1 (no significant disability, despite some symptoms) (Figure 7(b)). This result is consistent with previous studies that have reported correlation between FA in NAWM or in the tracts affected by stroke lesions and mRS in stroke patients [15, 83–86]. Our results specifically suggest that structural disruptions in *normal-appearing* WM are different depending on the level of disability and dependence in people with CVD. It should be noted that in the studied CVD cohort, there were only three subjects with an mRS of 3 (moderate disability) and only one subject with an mRS of 4 (moderately severe disability), which may explain why no difference was detected between NAWM FA in people with higher levels of disability compared to those with lower levels of disability.

Several limitations must be considered in the current study. First, the conventional DTI metrics FA and MD were utilized derived based on the single compartment diffusion tensor model [87]. Although previously shown to provide insight in numerous studies of development, aging, and pathological alterations caused by diverse neurological and neurodegenerative disorders, conventional DTI metrics may not accurately represent tissue microcompartments and their organization within the studied cerebral tissues, particularly where the WM tracts cross or the tissue microstructure is highly heterogeneous [28–30]. The DTI data in the current study were also acquired across 10 different scanners in Ontario. These data were acquired using the same diffusion pulse sequence, the same number of diffusion gradient directions, and the same magnetic field strength, which minimizes between-site variations [32]. Moreover, the DTIPrep QC protocol included as a quality control step in the image processing pipeline used for analysis of the DTI data [42] has previously been shown to significantly reduce variability in DTI measurements made across multiple scanners [47] and therefore contributed to the harmonization of the DTI data across sites in the current study. However, further harmonization of these data using more sophisticated meta- and mega-analysis statistical methods proposed for DTI data [88] may further improve the characterization of cerebrovascular lesions. Another limitation of this study relates to subjects who had multiple small lesions of the same type, especially multiple pWMHs. In such cases, very small lesions of the same type captured by the semiautomated segmentation procedure would be summed and meet the threshold for inclusion; however, DTI measurements in those small injuries may include partial volume errors. Finally, it should be noted that the current study quantified diffusion parameters in the chronic stroke setting. The results cannot be applied to acute stroke conditions, where diffusion parameter values are known to change over time, and perfusion/diffusion mismatch can identify penumbral tissue [89].

## 5. Future Directions

Future work should determine an ideal complement of quantitative diffusion biomarkers to identify vascular lesions and to predict tissue damage and consequent cognitive or functional decline.

## 6. Conclusions

This study characterized conventional DTI metrics FA, MD, AD, and RD in a cohort of 152 individuals with vascular lesions and found that FA and MD not only differed between vascular lesions and healthy tissue but also differed between vascular lesion subtypes. The variation in diffusion metrics observed within stroke regions and white matter hyperintensities provided unique contrast when compared to T1-weighted, T2-weighted, and FLAIR images. Consequently, the diffusion measures could improve the accuracy of tissue and vascular lesion classification using sophisticated multifeature image segmentation approaches and aid in the interpretation of imaging findings in people with CVD-related brain pathologies. This study also demonstrated an association between fractional anisotropy in normal-appearing white matter and hypertension in CVD subjects which highlights the key role of cerebrovascular disruption in promoting various neurodegenerative and neurological disorders. The association between normal-appearing white matter microstructure deterioration inferred by decreased fractional anisotropy and modified Rankin scale, a measure of degree of disability in stroke patients, also suggests that subtle global changes in normal-appearing white matter microstructure may impact the global disability outcome measure beyond the direct effects of vascular anomalies in people with CVD.

## Figures and Tables

**Table 1:** Concentration of blood components, blood pressure, and global cognition assessments for study participants (ONDRI CVD cohort). SD = standard division and MoCA = Montreal Cognitive Assessment.

	ONDRI CVD cohort
Number of subjects	152
Concentration of blood components (mmol/L) (mean ± SD)	71.3 ± 6.0
Glucose	6.0 ± 1.4
Triglyceride	1.3 ± 1.2
Cholesterol	3.8 ± 1.0
HDL cholesterol	1.3 ± 0.4
LDL cholesterol	1.9 ± 0.8
Blood pressure (mmHg) (mean ± SD)	
Systolic	133.7 ± 19.5
Diastolic	78.0 ± 10.2
Global cognition assessment (mean ± SD)	
MoCA	25.3 ± 3.0

**Table 2:** Values of the four major DTI metrics measured in the 10 global cerebral tissues/lesions considered in this study using the DTI processing pipeline. Mean, coefficient of variation (CV), and 95% confidence interval (CI) of the DTI metrics in each lesion were calculated across the ONDRI CVD subjects who have that specific tissue/lesion. Note that the first two rows provide the number and percentage of the subjects from the ONDRI CVD cohort (152 subjects in total) who have each type of these tissues/lesions. If the volume of a lesion within a subject's brain is smaller than 24 mm^3^, we assumed that the subject does not have that specific lesion type.

Tissue type		NAWM	dWMH	pWMH	PVS	NAGM	Stroke	pLACN	vCSF	dLACN	sCSF
# of subjects		152	145	152	116	152	86	85	152	56	152
% of subjects		100	95	100	76	100	57	56	100	37	100

FA	Mean	0.38	0.27	0.26	0.16	0.16	0.15	0.13	0.12	0.12	0.07
	CV	0.07	0.21	0.20	0.18	0.08	0.30	0.28	0.12	0.28	0.14
	95% CI	0.004	0.01	0.008	0.005	0.002	0.01	0.008	0.002	0.009	0.002

MD	Mean (mm^2^/s)	0.79	1.14	1.28	1.58	1.09	1.64	1.83	2.74	1.93	2.15
	CV	0.05	0.12	0.10	0.10	0.06	0.19	0.13	0.05	0.13	0.07
	95% CI (mm^2^/s)	0.006	0.023	0.021	0.03	0.011	0.066	0.053	0.02	0.069	0.023

AD	Mean (mm^2^/s)	1.13	1.47	1.63	1.85	1.25	1.86	2.07	3.07	2.16	2.30
	CV	0.04	0.09	0.08	0.09	0.06	0.16	0.12	0.04	0.11	0.07
	95% CI (mm^2^/s)	0.007	0.021	0.02	0.03	0.012	0.064	0.052	0.022	0.065	0.025

RD	Mean (mm^2^/s)	0.62	0.98	1.11	1.45	1.02	1.53	1.71	2.58	1.82	2.08
	CV	0.07	0.16	0.13	0.11	0.06	0.21	0.15	0.05	0.15	0.07
	95% CI (mm^2^/s)	0.007	0.026	0.023	0.03	0.011	0.068	0.055	0.02	0.071	0.023

## Data Availability

All the brain MR imaging data used in this study are available from a third party: the Ontario Brain Institute (OBI). Access to this data is managed by the OBI and the Ontario Neurodegenerative Disease Research Initiative (ONDRI). The process for gaining access to the data for eligible researchers is described in the Brain-CODE governance policy https://www.braincode.ca/content/about-brain-code. For more information, please refer to this website: https://ondri.ca/.

## Additional Points

*Code Availability*. Details describing the processing steps applied to the imaging data are described in this manuscript and also in a previously published manuscript related to the analysis of the ONDRI multisite diffusion data [42]. All the image processing procedures were completed using freely available image processing software packages such as FMRIB Software Library (FSL) and Advanced Normalization Tools (ANTs) [42]. All the statistical analyses related to the calculated diffusion tensor imaging metrics presented in this manuscript were performed using IBM SPSS (Version 26.0, Armonk, NY: IBM Corp). All other imaging data preparation, organization, rearrangement, and reformatting, as well as region of interest analyses of the diffusion data, were implemented in MATLAB (R2019b, Natick, Massachusetts: The MathWorks Inc.). All the related code is available upon request.
